# Social equity perception and public mental health: a Chinese study with panel data

**DOI:** 10.1186/s40359-023-01296-y

**Published:** 2023-09-05

**Authors:** Fan Yang, Yao Jiang, Xiu Bai, Yuchen Cai, Haiying Duan

**Affiliations:** 1 Department of Labor and Social Security, School of Public Administration, Sichuan University, Chengdu, China; 2 Department of Demography, Zhou Enlai School of Government, Nankai University, Tianjin, China; 3 School of Economics, Sichuan University, Chengdu, Sichuan China

**Keywords:** Social equity perception, Mental health, Happiness, Life satisfaction, Social trust, Depressive symptoms

## Abstract

**Background:**

Mental health is a vital part of an individual’s overall health and well-being, and the relationship between society and individuals has always been a focus of academic and public attention. However, the effect of social equity perceptions on individual mental health remains unclear.

**Methods:**

Data were collected from 8,922 survey respondents with an average age of 47.533 years from the China Labor-force Dynamics Survey 2016 and 2018. The Center for Epidemiological Studies Depression scale was used to assess mental health. A two-way fixed effects regression model was used to determine the association between social equity perception and individual mental health.

**Results:**

Individuals with higher perceptions of social equity were more likely to report better mental health ($\beta$ = -0.944, *p* < 0.01). Happiness, life satisfaction, and social trust partially play mediating roles in the relationship between social equity perception and individual mental health, while education and age play moderating roles.

**Conclusion:**

Social equity perception is a vital factor that affects mental health. Public policies should focus on helping less educated and older people improve their social equity perception to improve their mental health.

## Background

The relationship between society and individuals has always been a focus of academic and public attention [1–3]. The equity of a society may have a significant and far-reaching effect on individual experience, such as individual ethics [4], human rights, and health equity [5]. However, the effect of social equity perceptions on individual mental health remains unclear. This study explored this problem.

Mental health is a vital part of an individual’s overall health and well-being [6]. It generally refers to the condition of being mentally and emotionally sound, characterized by the absence of mental illness and adequate adjustment capability [7]. It specifically reflects feelings of comfort about oneself, positive feelings about others, and the ability to meet the demands of daily individual and social life [7]. Therefore, individual mental health involves relationships between individuals and themselves, individuals and others, and individuals and society.

Individuals need to believe that they live in a world where people generally obtain what they deserve [8]. In this paper, we suggest that the demand for social equity perception also affects individual mental health from the above-mentioned three aspects: the relationship between individuals and themselves, individuals and others, and individuals and society.

First, social equity perception may affect an individual’s mental health in terms of psychological harmony. Individuals with a high perception of social equity tend to have internal attributions rather than external attributions of their own negative outcomes, thus attenuating feelings of inequity and, in turn, leading to a decrease in negative emotions and achieving inner harmony [9], which is good for individual mental health [10]. Further, individuals with high social equity perception can also be expected to avoid excessive self-focused rumination, which is likely to protect their mental health [11]. In an emotional experiment, individuals high in social equity perception were less angry and suffered no decrease in self-esteem in the anger-evoking condition than individuals with low social equity perception, who reported increased feelings of anger and decreased self-esteem [11]. It can be concluded from the above emotional experiment that individuals with high social equity perception are better able to cope with anger-evoking situations and maintain psychological balance and harmony [11]. Therefore, social equity perceptions can be regarded as a personal resource that reduces negative emotions, avoids excessive introspection, maintains inner harmony, and protects mental health.

Second, social equity perceptions may affect individual mental health in terms of harmony with others. To a certain extent, individuals that believe the world treats them fairly are more likely to view certain traits in others as diverse and flexible and are open to changing themselves when necessary [12]. Therefore, they become more receptive to the qualities, personalities, and habits of others, making it easier to get along with others [12]. In addition, social equity perception endows individuals with the confidence that they can be treated fairly by others and do not suffer from an unforeseeable disaster [11]. Therefore, individuals with a higher perception of social equity generally have more trust in others [13] and are more tolerant of the complex and heterogeneous prosocial behavior of others [14], which benefits their mental health.

Third, social equity perceptions may affect individual mental health through social trust. Social trust in the public is not an isolated social-psychological phenomenon. This is related to interpersonal trust, social security, social recognition, government satisfaction, social evaluations, and social attitudes. However, among all these factors, the key factor underlying social trust is the perception of equity in society [15], which means that there might be a close relationship between social equity perception and social trust. According to an assessment in 2015, there was a moderate positive correlation between perceived social equity and social trust [16]. This indicates that an improvement in social equity is conducive to the improvement of social trust. The Blue Book of Social Mentality: Annual Report on Social Mentality of China 2016, released by the Social Psychology Research Center of the Institute of Sociology of the Chinese Academy of Social Sciences, pointed out that in today’s Chinese society, the perception of social equity has become a vital source of trust in civil relations, which means that the more people feel that society is fair, the more they trust this society [17]. Further, several studies have shown that individual social trust is inversely related to depressive symptoms [18, 19]. Higher social trust cognition can reduce the risk of major depression [20]. In addition, generalized social trust as social capital can be associated with positive mental health [21]. Similarly, a study showed that in the same community, individuals who expressed a high level of social trust also had a higher self-rated health degree [22]. The above studies indicate that social equity perception may affect individual mental health through social trust.

In summary, although the existing literature theoretically predicts that there may be a close relationship between social equity perception and individual mental health, the relationship between the two has not been widely verified, especially in rapidly developing economies and developing countries. China is currently experiencing rapid economic development. At the same time, social members hope that the wealth of economic development can be distributed more equitably. Distribution equity reform has been put on China’s national agenda [23]. Therefore, it is of great practical significance to explore the social equity perception of the public and its influence in China, a society with the rapid growth of wealth.

Accordingly, the objective of this study was to understand how social equity perception affects individual mental health in a country with rapid economic development and uneven income distribution. For this purpose, we employed 8,922 respondents aged between 18 and 81 years from the China Labor-Force Dynamics Survey (CLDS) 2016 and 2018. A two-way fixed effects regression model was used to determine the association between social equity perception and individual mental health.

Our study expands the body of knowledge on the relationship between individuals and society by focusing on the effect of social equity perceptions on individual mental health. We observed a significant effect of social equity perception on individual mental health using nationally representative Chinese panel data. In addition, we explored the mediating roles of happiness, life satisfaction, and social trust as well as the moderating roles of education and age in the influence of social equity perception on individual mental health. Previous theoretical analysis has shown that social equity perception may affect individual mental health through harmony with oneself and others and social trust. Generally, maintaining harmony between oneself and others is closely related to happiness and life satisfaction. The more harmonious individuals are with themselves and others, the more likely they are to feel happy, and the more satisfied they are with life [10, 24]. Therefore, we applied happiness and life satisfaction to express the harmony between oneself and others. Our study should be of interest to public institutions aiming to promote social equity as well as relevant researchers and the public pursuing social equity.

## Methods

### Data

The data employed in this study were obtained from two waves of data (2016 and 2018) of the CLDS. The CLDS is a comprehensive survey organized by the professional institution of a famous university (the Center for Social Science Survey at Sun Yat-sen University, a Chinese university with a global reputation). The data of CLDS 2016 and 2018 were collected from 29 provincial administrative units across the country. Therefore, the data are nationally representative. The data of CLDS 2016 and 2018 collected in the survey included the objective conditions of the respondents, such as education, occupation, migration, health, and economics, as well as their subjective perceptions of some factors, phenomena, and atmosphere. The CLDS used a multistage cluster and stratified probability-proportional-to-size sampling strategy. The investigators collected data from respondents through door-to-door or telephone interviews. The CLDS 2016 collected data from 21,086 respondents, and based on the CLDS 2016, the CLDS 2018 conducted a followed-up survey. Finally, after cleaning the data of the CLDS 2016 and 2018 by excluding outliers and missing values, we obtained useful samples of 8,922 respondents who were matched across the CLDS 2016 and 2018.

### Measures

#### Explained variable

The variable explained in this study was individual mental health. The Center for Epidemiological Studies Depression (CES-D) scale was used to measure the respondents’ mental health. The CES-D scale developed by Radloff is one of the most widely used scales in the world for measuring depression and mental health [25], and its effectiveness in mental health measurements has been confirmed by a large number of studies [26, 27]. The CES-D scale has 20 items scored from 20 to 80. A lower score indicated a lower level of depression and better mental health [25].

#### Explanatory variable

The explanatory variable we focus on in this study is individual social equity perception, and it is a continuous variable. Respondents were asked, “How would you evaluate the recent overall social equity situation?” and rated their responses from 1 to 5 on a five-point Likert scale that included “very inequitable,” “somewhat inequitable,” “normal,” “somewhat equitable,” and “very equitable.” Except for measuring social equity perception by considering it as a continuous variable, to test the robustness of the empirical results, in the section of robustness checks we also treated social equity perception as a categorical variable.

#### Mediating variable

The three mediating variables considered in this study are happiness, life satisfaction, and social trust. For happiness, respondents were asked, “How would you evaluate your happiness?” and rated their responses from 1 to 5 on a five-point Likert scale that included “very unhappy,” “somewhat unhappy,” “normal,” “somewhat happy,” and “very happy.”

For life satisfaction, respondents were asked, “Are you satisfied with your current life?” and could rate their responses from 1 to 5 on a five-point Likert scale that included “very dissatisfied,” “somewhat dissatisfied,” “normal,” “somewhat satisfied,” and “very satisfied.”

For social trust, respondents were asked, “In general, do you agree that most people in society can be trusted?” The responses included “strongly disagree,” “somewhat disagree,” “normal,” “somewhat agree,” and “strongly agree,” and were rated from 1 to 5 on a five-point Likert scale.

#### Control variables

To adjust for potential confounding effects on the relationship between social equity perception and individual mental health, we controlled for several variables that may have affected individual mental health in our regression model analyses. These variables included age, education, marital status [28], income [29], smoking [30], drinking [31], exercise [32], and self-reported physical health [33]. The detailed definitions of these variables are presented in Table 1.

**Table 1:** Variable definition and descriptive statistics (*n* = 8,922)

**Variable**	**Definition**	**Mean**	**SD**	**Min**	**Max**
**Explained variable**					
Mental health	Total score of the CES-D ranges from “20” to “80”. The higher CES-D score, the deeper depression, and the worse mental health	27.392	8.831	20	80
**Explanatory variable**					
Social equity perception	1 = very inequitable; 2 = somewhat inequitable; 3 = normal; 4 = somewhat equitable; 5 = very equitable	3.276	0.929	1	5
**Control variable**					
Age	Years old	47.533	11.177	18	81
Education	Years of schooling education	8.275	4.125	0	23
Marital status	1 = married; 0 = unmarried	0.912	0.283	0	1
Logarithm of income	Logarithm of total annual income of respondent in 2017	9.864	1.204	3.689	14.221
Smoking	1 = have habit of smoking; 0 = else	0.335	0.472	0	1
Drinking	1 = have habit of drinking; 0 = else	0.237	0.425	0	1
Exercise	1 = have habit of exercise; 0 = else	0.282	0.450	0	1
Self-reported physical health	1 = very bad; 2 = somewhat bad; 3 = normal; 4 = somewhat good; 5 = very good	3.571	0.978	1	5

### Analysis strategy

The explained variable in this study is individual’s mental health which is a continuous variable. To examine the association between social equity perception and individual mental health, while avoiding the problem of omitted time-invariant and year shift unobservable factors, we rely on the following two-way fixed effects ordinary least squares model:

1 $$Mental\;{health}_{i,t}=\alpha+{\beta SEP}_{i,t}+{yZ}_{i,t}+\mu_i+{year}_t+\varepsilon_{i,t}$$

where ${\text{Mental health}}_{\text{i,t}}$ is the explained variable, the mental health status of respondent* i* in year *t*, which was measured by the CES-D scale; ${\text{SEP}}_{\text{i,t}}$ is the explanatory variable, namely, social equity perception of respondent *i* in year *t*; ${\text{Z}}_{\text{i,t}}$ represent the set of control variables (i.e., age, education, marital status, income, smoking, drinking, exercise, and self-reported physical health); $\beta$ and γ are the coefficient vectors; $\alpha$ is the intercept term; ${\mu }_{\text{i}}$ and ${\text{year}}_{\text{t}}$ are the fixed effects for *i* respondent and *t* survey year, respectively.

## Results

### Descriptive statistics

The definitions of the variables employed in this study and the results of the descriptive analysis (*n* = 8,922) are reported in Table 1. Regarding the explained variable, the average value for respondents’ mental health was 27.392 (SD = 8.831) on the CES-D scale ranging from 20 to 80. This means that the overall depression value of the respondents was low and their mental health level was good on average.

The average value of the explanatory variable — social equity perception — is 3.276 (SD = 0.929) on the five-point Likert scale ranging from 1 to 5, which means the social equity perception of the respondents was generally between “normal” and “somewhat equitable.” This indicates that the respondents’ overall social equity perception was not relatively high, and there is room for improvement.

Regarding the control variables, the ages of the respondents ranged from 18 to 81 years, with a mean value of 47.533 (SD = 11.177). The average number of years of schooling was 8.275 (SD = 4.125), which is close to the nine-year compulsory education level implemented in China. The logarithm of the total annual income of the respondents had a mean value of 9.864 (SD = 1.204), with a minimum value of 3.689 and a maximum value of 14.221. A total of 91.2% of the respondents were married, 33.5% smoked, 23.7% drank alcohol, and 28.2% exercised regularly. The mean value of respondents’ self-reported physical health was 3.571 (SD = 0.978), which ranged from 1 to 5.

### Fixed effects regression

Table 2 reports the two-way fixed effects model regression results of the effect of social equity perception on individual mental health. In column (1) of Table 2, when only the variables of social equity perception and fixed effects of individual and year are in the model, social equity perception significantly affects individual mental health at the significance level of 1% ($\beta$ = -1.152). In column (2), when fixed effects and variables of age, education, marital status, and the logarithm of income are added to the model, the social equity perception still significantly affects individual mental health at the significance level of 1% ($\beta$ = -1.150). In column (3), when fixed effects and all the control variables are added to the model, the social equity perception still significantly affects individual mental health at the significance level of 1% ($\beta$ = -0.944). The above results indicate that the higher the individual social equity perception, the better their mental health. Therefore, the preliminary conclusion can be drawn that social equity perception does significantly affect individual mental health, as posited in the previous theoretical analysis.

**Table 2:** Influence of social equity perception on individual mental health by using ordinary least squares model (fixed effects)

**Variable**	**(1)**	**(2)**	**(3)**
Social equity perception	-1.152***	-1.150***	-0.944***
	(0.269)	(0.265)	(0.246)
Age		0.075	0.102
		(0.090)	(0.079)
Education		-0.055	-0.022
		(0.095)	(0.091)
Marital status		-0.690	-0.597
		(1.439)	(1.382)
Logarithm of income		-0.046	-0.099
		(0.205)	(0.192)
Smoking			-0.486
			(0.485)
Drinking			0.292
			(0.281)
Exercise			-0.113
			(0.389)
Self-reported physical health			-1.792***
			(0.142)
Fixed effects			
Individual	Yes	Yes	Yes
Year	Yes	Yes	Yes
*n*	8,922		
R^2^	0.655	0.655	0.667

Robust standard errors in parentheses

*** Significance level at 1%

“Yes” means the variable is added to the model

### Robustness check

#### Sub-group regression

To test the robustness of the previous regression results, we conducted a sub-group regression based on the gender of respondents using the two-way fixed effects ordinary least squares model, and the results are reported in Table 3. From the regression results of the subgroups in Table 3, the influence of social equity perception on individual mental health is significant among the subgroups of males ($\beta$ = -1.015) and females ($\beta$ = -0.843). These results indicate that the influence of social equity perception on individual mental health is stable. This confirms the previous finding that social equity perception is a vital factor in the mental health of individuals.

**Table 3:** Sub-group regression of influence of social equity perception on individual mental health by using ordinary least squares model (fixed effects)

**Variable**	**Gender**	
	**(1) Male**	**(2) Female**
Social equity perception	-1.015***	-0.843**
	(0.293)	(0.317)
Control variables	Yes	Yes
Fixed effects		
Individual	Yes	Yes
Year	Yes	Yes
*n*	4,876	4,046
R^2^	0.647	0.680

Robust standard errors in parentheses

***, ** Significance level at 1 and 5%, respectively

“Yes” means the variable is added to the model

#### Changing the measurement of explanatory variable

In addition to subgroup regression, we also considered social equity perception as a categorical variable and employed the two-way fixed effects ordinary least squares model for regression. The results of changing the measurement of the explanatory variable were presented in Table 4. It can be observed from column (2) of Table 4 that using response of “very inequitable” as a reference, the coefficients of “somewhat inequitable” ($\beta$ = -1.874), “normal” ($\beta$ = -2.742), “somewhat equitable” ($\beta$ = -3.701), and “very equitable” ($\beta$ = -3.664) are all significant. The results indicate that, compared to a lower level of social equity perception, a higher level of social equity perception has a stronger effect on individuals’ mental health. Therefore, although the measurement of social equity perception is changed, the results that social equity perception has a significant influence on individuals’ mental health are still held.

**Table 4:** Results of changing the measurement of explanatory variable by using ordinary least squares model (fixed effects)

**Variable**	**(1)**	**(2)**
Very inequitable as reference		
Somewhat inequitable	-2.047*	-1.874*
	(1.102)	(1.063)
Normal	-3.102**	-2.742**
	(1.166)	(1.098)
Somewhat equitable	-4.245***	-3.701***
	(1.381)	(1.291)
Very equitable	-4.735***	-3.664**
	(1.468)	(1.324)
Control variables		Yes
Fixed effects		
Individual	Yes	Yes
Year	Yes	Yes
*n*	8,922	8,922
R^2^	0.655	0.667

Robust standard errors in parentheses

***, **, * Significance level at 1, 5, and 10%, respectively

“Yes” means the variable is added to the model

### Exploring the mediating roles of happiness, life satisfaction, and social trust

Previous theoretical analysis shows that social equity perception may affect individual mental health through harmony with oneself and others and social trust. In this section, we examine the mediating roles of harmony with oneself and others and social trust. As pointed out in the introduction, we use happiness and life satisfaction to express harmony with oneself and with others. The results are presented in Table 5.

**Table 5:** Mediating effects of happiness, life satisfaction, and social trust in effect of social equity perception on individual mental health (fixed effects)

**Variable**	**(1) Mental health**	**(2) Happiness**	**(3) Mental health**	**(4) Life satisfaction**	**(5) Mental health**	**(6) Social trust**	**(7) Mental health**
Social equity perception	-0.944***	0.139***	-0.774***	0.174***	-0.738***	0.037***	-0.926***
	(0.246)	(0.018)	(0.221)	(0.017)	(0.227)	(0.009)	(0.237)
Happiness			-1.229***				
			(0.183)				
Life satisfaction					-1.186***		
					(0.168)		
Social trust							-0.491
							(0.456)
Control variables	Yes	Yes	Yes	Yes	Yes	Yes	Yes
*n*	8,922						
R^2^	0.667	0.660	0.672	0.674	0.672	0.601	0.667

Robust standard errors in parentheses

*** Significance level at 1%

“Yes” means the variable is added to the model

As shown in Table 5, social equity perception has a significant effect on happiness (column 2, coefficient = 0.139), life satisfaction (column 4, coefficient = 0.174), and social trust (column 6, coefficient = 0.037) at a significance level of 1%. Furthermore, when we added happiness, life satisfaction, and social trust separately to the model, the absolute value of the effect coefficient of social equity perception on individual mental health was less than when each of these three variables was not added (|-0.774| < |-0.944|; |-0.738| < |-0.944|; |-0.926| < |-0.944|). This indicates that happiness, life satisfaction, and social trust mediate the effect of social equity perception on individual mental health [34].

### Exploring the moderating roles of education and age

**Fig. 1:**
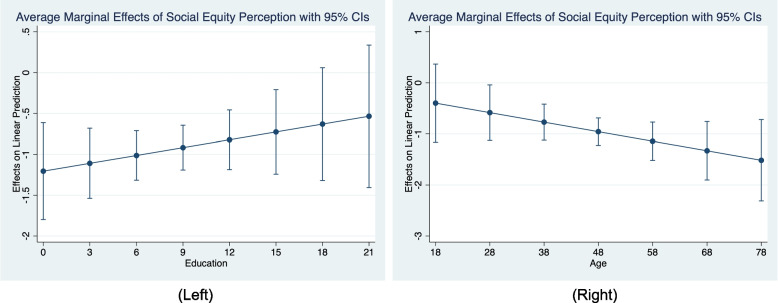
The average marginal effect of social equity perception on individual mental health with different education and age

In this section, we explore the moderating roles of education and age in the effect of social equity perception on individual mental health. The left side of Fig. 1 shows the average marginal effects of social equity perception on individual mental health with different levels of education. The results show that the fewer years of education, the greater the absolute value of the average marginal effect of social equity perception on individual mental health. This indicates that education is a vital moderator of the effect of social equity perception on individual mental health. Further, it reveals that the social equity perception of individuals with lower education has a greater effect on their mental health than that of individuals with higher education.

The right side of Fig. 1 shows the average marginal effects of social equity perception on individual mental health at different ages. The results showed that the older the age, the greater the absolute value of the average marginal effect of social equity perception on individual mental health. This indicates that age is also a significant moderator of the effect of social equity perception on individual mental health. Furthermore, this means that the social equity perception of older individuals has a greater effect on their mental health. In other words, the mental health of older people depends more on their social equity perception than that of younger people.

## Discussion

An individual is nested in society, and the individual and society influence each other. Individual actions shape social form. Social forms also have a far-reaching effect on individual thoughts, behaviors, and states. This study found that social equity perception had a significant effect on individual mental health. Specifically, individuals with higher social equity perceptions are more likely to report better mental health. Further, we found that happiness, life satisfaction, and social trust partially mediated the relationship between social equity perception and individual mental health. Additionally, individuals’ education and age moderated the effect of social equity perception on mental health.

The relationship between individuals and society is an old and often a new topic. Previous studies have explored the crucial influences of factors at the material level on individuals’ mental health such as income [35] and built environment [36]. The far-reaching effects of social equity on individuals’ experience, incorporating individual ethics [4], human rights, and health equity [5] also have been discussed in the existing literature. However, the influence of social equity perception on individuals’ mental health is under-explored. By investigating the effect of social equity perception, a factor at the individual spiritual level, on people’s mental health, this study enriches research on exploring the factors affecting individual health and the relationship between individual health and their social attitude [4, 5]. The equity of a society is shaped, formed, and perfected by its individuals. This is the role played by individuals in social equity. However, the equity of a society and individuals’ perceptions of it in turn has a series of profound effects on individuals. This study has found that the perception of social equity significantly affects individuals’ mental health. Therefore, it is not only a political issue but also a public health policy and management issue to strive to build a more equitable society and continuously improve individuals’ perceptions of social equity. The equity of society depends on the rational design of a series of social systems. It requires full respect for all kinds of professionals with professional knowledge as well as systematic and overall thinking. At the same time, systems that promote social equity should not dampen people’s enthusiasm to strive for efficiency.

The mechanism analysis has shown that the influence of social equity perception on individual mental health is mediated by their happiness, life satisfaction, and social trust. It may be explained that individuals who have higher social equity perception may undertake higher levels of happiness, life satisfaction, and social trust, and there are higher possibilities that individual happiness, life satisfaction, and social trust can exert positive influences on their mental health status. Happiness, life satisfaction, and social trust are important determinants for people’s mental health, and in previous studies, they are also routinely employed as indicators of individual societal well-being outcomes [37–39]. Especially, during the coronavirus disease (COVID-19) pandemic, the lockdown measures present serious adverse effects on people’s emotional well-being worldwide [40]. Many studies were centered on people’s happiness, life satisfaction, and social trust, and demonstrated the significant roles that happiness, life satisfaction, and social trust played for improving individual mental health in the environment of lockdown measures [41–43]. Collectively, the empirical results of the mediation test are consistent with our theoretical analysis and previous studies.

The policy of improving the perception of social equity should give preference to vulnerable groups. In this study, we found that the perception of social equity had a greater effect on the mental health of individuals with lower education and older people. People with low education levels are often at the bottom of society, and older people are more dependent on social security than younger people. They are more likely to experience social inequity [44]. Therefore, social policies to improve social equity should be more inclined towards these groups. For example, the government can provide free vocational training and internship opportunities to less-educated individuals to promote their vocational skills and human capital and offer necessary social relief for low-income people when they need it to let them feel the warmth of society. The public sector can provide low-income individuals with income incentive plans in the form of tax deductions or tax exemptions to help them increase their income while fully respecting their dignity. This is not only conducive to improving the perception of social equity among such vulnerable groups but is also beneficial to their mental health.

The results of this study have a practical significance in terms of achieving common prosperity for all citizens in China, which has always been the unremitting pursuit of the Chinese people and the Chinese government. Social equity is undoubtedly conducive to achieving common prosperity. Although China’s social equity is constantly improving, there is still potential for more. For example, China still has room for improvement in the fair distribution of educational opportunities and medical resources [45, 46]. The fair distribution of these social public resources helps to enhance individuals’ perception of social equity to a large extent, making them believe that they live in a fair society, boosting their happiness, life satisfaction, and social trust, and contributing to their mental health, which is undoubtedly the key consideration and concern for the common prosperity of a society.

This study has two limitations that should be mentioned. First, the perception of social equity is a highly subjective variable, and this study only used the answer to a question to measure it. If a scale can be developed to measure it in future research, more accurate results may be obtained. Second, in this study, research on the promotion strategy of social equity perception is not in-depth, which is a meaningful direction for future research in this field.

## Conclusions

Employing two waves of nationally representative data from 8,922 respondents, this study explored the relationship between individual mental health and social equity perception. We found that individuals with higher perceptions of social equity were more likely to report better mental health. Happiness, life satisfaction, and social trust partially played mediating roles. Moreover, education and age played moderating roles in the effect of social equity perception on individual mental health. Therefore, as a public health policy and management issue, improving the degree of social equity and enhancing the public’s perception of social equity are of great importance for individual mental health. Formulating strategies to improve social equity could be a key breakthrough in further research and practice.

## Data Availability

As the data employed in this study come from public databases (Application Email: cssdata@mail.sysu.edu.cn), we got the administrative permissions to access the raw data used in this study from the Center for Social Science Survey at Sun Yat-sen University.

## Abbreviations

CLDS: China Labor-force Dynamics Survey
CES-D: The Center for Epidemiological Studies Depression
